# Lead (Chemicals and Contaminants)

**DOI:** 10.14252/foodsafetyfscj.D-22-00004

**Published:** 2022-06-24

**Authors:** Food Safety Commission of Japan

## Abstract

Food Safety Commission of Japan (FSCJ) conducted risk assessment of lead (hereinafter referred to as Pb) as a Self-Tasking assessment. Risk assessments of Pb were initially requested from the Ministry of Health, Labour and Welfare in relation to revision of the standards for apparatus/container and packaging (ACP) and to revision of the standards for beverages. Considering the diverse modes and routes of human exposure to Pb, FSCJ judged that a comprehensive risk assessment of Pb is appropriate instead of specific risk assessment relating to ACP or beverages. To estimate the actual Pb exposure of the general population in Japan, the present Self-Tasking assessment working group started to inspect available data of blood Pb levels among children (12 years old boys and girls, n = 289, surveyed for 2015-2018) and among adults (pregnant women, n = 96,696, surveyed for 2011-2014)^1)^. FSCJ concluded that average blood level of Pb of current Japan is about 1 µg/dL or less based on the data available at present. Comprehensive evaluation of the findings from the previous epidemiological studies suggested that even blood Pb at the level of 1-2 µg/dL potentially affected children’s neuro-behavioral development or adult renal function. FSCJ, however, concluded that figuring out of a blood Pb level without adverse effects was difficult from the data of epidemiological studies. Pb level in current Japan as about 1 µg/dL or less. This value is close to the level potentially to have some effects, 1-2 µg/dL as suggested by epidemiological studies. Continuous implementation of measures to reduce Pb exposure is thus required. A close watch on the trend of blood Pb level by human biomonitoring is also necessary for verification of the efficacy of the measures to reduce Pb exposure.

## Conclusion in Brief

Food Safety Commission of Japan (FSCJ) conducted risk assessment of lead (hereinafter referred to as Pb) as a Self-Tasking assessment. Risk assessments of Pb were initially requested from the Ministry of Health, Labour and Welfare in relation to revision of the standards for apparatus/container and packaging (ACP) and to revision of the standards for beverages. Since Pb occurs ubiquitously in the environment, the exposure sources are the medium including ambient air, soil, water and house dust in addition to food and water. Historically, most of the human studies have described the health effects with relation to the blood lead level of the subjects, which is a qualified bio-maker of exposure to Pb. Considering the diverse modes and routes of human exposure to Pb, FSCJ judged that a comprehensive risk assessment of Pb is appropriate instead of specific risk assessment relating to ACP or beverages.

Pb-containing substances originate widely in the natural environment, and also in anthropogenic sources. Consequently, humans are exposed to Pb in the daily life from a wide variety of sources including meals (exposure not only through food but also drinking water and lead-contaminated ACP), atmosphere, soil, house dust, and the others. Findings on relative contribution of each source for the exposure are, however, inconsistent with studies to studies. Moreover, no clear trend is found on a specific food group with the substantial contribution. The daily intake of Pb from the market basket surveys were more than 100 µg/day in 1978 in Japan. The estimated daily intake of Pb from meals continued to sharply decrease by 1982, and then kept the same trend afterward. The recent values were reported as 2 to 9 μg/day in the surveys using the market basket method and also the duplicate diet method.

Blood Pb level is recognized to reflect the actual human Pb exposure from various sources, including meals. Blood Pb level thus has been used as the exposure marker widely in epidemiological studies on the effects of chronic exposure to Pb.

To estimate the actual Pb exposure of the general population in Japan, the present Self-Tasking assessment working group started to inspect available data of blood Pb levels among children (12 years old boys and girls, n = 289, surveyed for 2015-2018) and among adults (pregnant women, n = 96,696, surveyed for 2011-2014)^1^^)^. The children were subjects of the Tohoku Study of Child Development and the adults were subjects of the Japan Environment and Children’s Study. As a result, the median and 95 percentile of blood Pb level in the children of 12 years old were calculated as 0.66 µg/dL and 1.04 µg/dL, respectively. The median and 95 percentile of blood Pb level in pregnant women were 0.61 µg/dL and 1.11 µg/dL, respectively. It should be noted that, since the data are from a limited number of an age group and of a local area, the children’s blood Pb level do not necessarily reflect children as a whole over Japan. There is also uncertainty in presuming the blood Pb level of all adults in Japan based on the level in pregnant women only. Since pregnant women may have blood Pb levels lower than non-pregnant women and furthermore age difference and gender difference in blood Pb level have been observed in surveys abroad.

Estimation of blood Pb level of all Japanese is difficult due to the lack of surveys with the representative sample where gender, age groups, and areas were considered, as described above. Nonetheless, FSCJ concluded that average blood level of Pb of current Japan is about 1 µg/dL or less based on the data available at present, in considering the above-mentioned uncertainty. In addition, the blood Pb level of Japanese decreased in comparison to that in 1990s, and the current level was judged to be rather low among countries in the world.

Many models have been developed to calculate mathematically Pb intake from blood Pb level. There are, however, various problems in every model. The results to support the relationship between blood level and intake of Pb are insufficient and yet to be proven. FSCJ thus concluded that the conversion of a blood Pb level into a tolerable daily intake of Pb is difficult.

To estimate continuously the actual Pb exposure from different exposure sources, including meals, it is necessary to watch closely the trend of blood Pb level with representative samples through implementations of human biomonitoring with substantial sizes in Japan, like the case conducted over the world.

FSCJ tried to specify a blood Pb level without showing any adverse effect using data from epidemiological studies. Comprehensive evaluation of the findings from the previous epidemiological studies suggested that even blood Pb at the level of 1-2 µg/dL potentially affected children’s neuro-behavioral development or adult renal function. FSCJ, however, concluded that figuring out of a blood Pb level without adverse effects was difficult from the data of epidemiological studies. The following reasons were considered. 1) some inconsistency were observed among epidemiological studies, 2) difficulty to eliminate confounding factors completely, 3) insufficient amounts of evidence for providing a causal relationship between Pb exposure and the observed effect, 4) clinical significance and public health significance of the observed effects remained obscure. 

FSCJ estimated, on the basis of the above findings, Pb level in current Japan as about 1 µg/dL or less. This value is close to the level potentially to have some effects, 1-2 µg/dL as suggested by epidemiological studies. Continuous implementation of measures to reduce Pb exposure is thus required.

Note that this assessment was conducted based on the currently available scientific findings. To conduct risk assessment more precisely, it is desirable to accumulate scientific findings such as data on Pb exposure from various mediums and of blood Pb level, and also scientific findings on the effects of the low level Pb exposure in Japanese people.

A close watch on the trend of blood Pb level by human biomonitoring is also necessary for verification of the efficacy of the measures to reduce Pb exposure.
